# Exploring the Potential Health Risks Faced by Waste Pickers on Landfills in South Africa: A Socio-Ecological Perspective

**DOI:** 10.3390/ijerph16112059

**Published:** 2019-06-11

**Authors:** Catherina J Schenck, Phillip F Blaauw, Jacoba MM Viljoen, Elizabeth C Swart

**Affiliations:** 1 DST/NRF/CSIR Chair in Waste and Society, University of the Western Cape, Bellville 7535, South Africa; 2 School of Economic Sciences, North-West University, Potchefstroom Campus, Potchefstroom 2520, South Africa; derick.blaauw@nwu.ac.za; 3 School of Economics and Econometrics, University of Johannesburg, Johannesburg 2006, South Africa; kotiev@uj.ac.za; 4 Department of Dietetics and Nutrition, University of the Western Cape, Bellville 7535, South Africa; rswart@uwc.ac.za

**Keywords:** waste pickers, landfill, landfill waste picker, recyclables, health risks, South Africa

## Abstract

Landfill and street waste pickers in South Africa are responsible for collecting substantial volumes of recyclable material, saving municipalities millions and contributing to a generally healthier and cleaner environment. Yet waste pickers continue to operate on the fringes of the economy and are exposed to many risks, particularly health risks which have a direct impact on the sustainability of their livelihoods. This article, using a mixed-methods approach, explores the health risks to which waste pickers working on nine different landfills in the country are exposed. The socio-ecological framework was used to analyse and present the results. A key finding was that waste picking, by its very nature, lends itself to innumerable health risks, but that these can be lessened through concerted and collaborative efforts on the part of landfill operators, local authorities and other stakeholders. Integrating the ‘self-employed’ waste pickers into the formal waste management system should be comprehensive in order to limit health risks. Waste pickers will never have a risk-free environment, but facilitative policies and supportive institutions can collaboratively help to mitigate these risks and create a more sustainable and dignified working environment towards sustaining their livelihoods.

## 1. Introduction

The United Nations Environmental Programme [1] refers to waste pickers as the ‘invisible environmentalists’ of the world. Waste pickers depend on collecting recyclable and other materials from the streets and landfills for their livelihoods [2]. Although operating informally, waste pickers are responsible for the recycling of as much as 50% of the plastic waste in the world [1]. In Brazil, waste pickers collect 92% of the country’s aluminium for recycling and 80% of the cardboard boxes [3], affording municipalities significant cost savings in the maintenance of landfill airspace. Their actions also help to protect natural resources and reduce air pollution and greenhouse gas emissions. Despite engaging in dirty and hazardous work, waste pickers contribute to a country’s economy and can help to promote the health and well-being of society. In fact, they contribute to most, if not all, of the United Nations (UN) Sustainable Development Goals (SDGs) [4]. Yet waste pickers still operate on the fringes of the formal waste system, risking their own health in their efforts to be self-reliant and to make a decent living for themselves. 

In the South African context, street and landfill waste pickers divert 90% of the 10% of waste that is recycled away from the country’s landfills [5]. Waste pickers also divert non-recyclable but re-usable goods, such as bricks, wood, furniture, household goods and food [6,7,8] away from the landfills. Despite the contribution from the waste pickers, 90% of waste is still dumped on the 876 landfills that are spread across South Africa [9]. This theoretically presents opportunities for more people to earn an income recovering recyclables. The South African government and the formal waste sector have to an increasing extent been recognising the important role that waste pickers play in diverting waste from the landfills [5]. In fact, diverting waste from the landfills has become one of the major priorities of the Department of Science and Technology (DST) and Council for Scientific and Industrial Research (CSIR) Research, Development and Innovation Road Map [10], an initiative in which waste pickers have the potential to play an even bigger role.

Currently attention is being given to ways and means of integrating waste pickers into the formal waste management system in South Africa. The success of the envisaged integration process is dependent on increasing the value placed on waste pickers’ work, facilitating greater access to waste, acknowledging that waste pickers’ voices are just as important as those of other role players in the waste management system, improving waste pickers’ earning potential and income, and improving their health through improved working conditions [11,12].

It is estimated that there are between 60,000 and 90,000 waste pickers on the landfills and streets of South Africa, but with rising unemployment and urbanisation, there may actually be as many as 215,000 [5]. Uncontrolled urbanisation in developing countries leads to an increase in urban poverty and inequality and more people resorting to informal economic activities [13]. Amegah and Jaakkola (2016) regard sub-Saharan Africa as the fastest urbanising region in the world, with the fastest growing poverty trend [13]. 

A critical but neglected area of research both globally [13,14,15,16] and locally in South Africa [8,11,17] is the health risks that waste pickers are exposed to. If waste pickers were formally employed at municipalities and waste companies, they would be protected by occupational, health and safety regulations. Being informal workers, they have no such protection [18]. 

According to Cointreau (2006), the handling of waste has risk implications for many stakeholders—from the household that disposes of the waste to waste pickers and formal waste workers who collect, sort and recycle or dispose of the materials on the landfills [16]. For most formal waste workers, precautionary measures are taken by their employers, such as providing protective clothing, training and washing facilities. The informal waste workers who are directly exposed to the waste have the least protection against health risk issues. While formal waste workers’ contact with the waste comes from picking up bags and bins, the waste pickers extract recyclable material directly from mixed dry and wet waste bags and open landfill dumps. In more developed countries, formal waste workers are only indirectly involved with the waste, working in mechanised sorting facilities and wearing protective clothing. Informal waste pickers in developing countries do not enjoy formal occupational, health and safety protection.

Clearly, despite the contribution of waste pickers to recycling efforts and waste diversion from landfills, the nature of their work presents many social, economic and health risks [1,2,8,14,16,19,20,21,22]. Only two studies in South Africa have focused on the health of waste pickers on landfill sites. One study was conducted in Durban [23] and the other in Tshwane [24]. This article extends the scope and depth of the existing literature by highlighting the health risks to which waste pickers are exposed on nine different landfill sites in South Africa. 

Internationally, some attention has been given to the health risks of waste workers, including waste pickers [1,16,21,25,26]. These studies found that waste pickers are concerned about their health, mainly because if their health is compromised it could prevent them from working and earning an income to support themselves and their families. 

However, the health risks faced by waste pickers should be viewed within the broader context of their living and working environments. Cointreau (2006) and Gutberlet and Baeder (2008) allude to the fact that some of the waste pickers’ health issues are not only due to their working with waste without the necessary protection [16,21]. They are also a by-product of their poor living conditions, evidenced in insufficient water, a lack of sanitation facilities, polluted air and food insecurity if they live in informal housing settlements close to the landfill [21]. Living on or next to the landfill or in informal, under-resourced housing units or shelters intensifies the exposure to health risks. 

The International Labour Organization [27] regards health and safety in the workplace as the promotion and maintenance of the highest degree of physical, mental and social well-being of workers in all occupations. The World Health Organization (WHO) [28] views workers’ health as being vulnerable to factors that could lead to cancers, accidents, musculoskeletal diseases, respiratory diseases, hearing loss, circulatory diseases, stress-related disorders, communicable diseases, and others. The WHO (2019) also states that working conditions, both in the formal and informal economy, bring other important factors into play, including working hours, salary, workplace policies concerning maternity leave, and provisions relating to the promotion and protection of workers’ health [28].

In addition, health and environmental rights require that a person’s health should not suffer because of the environment in which they live, work, play and learn. Provisions for the health and safety of workers are seen to make a major contribution to the well-being of an employee. In practice, a focus on health and safety involves the assessment of risks to which people are exposed and the modification of systems to mitigate or eliminate the risks [15,28]. 

Exploring some of the health risks and vulnerabilities experienced by waste pickers on nine landfills sites in South Africa—which this article sets out to do—is a valuable step in the process of building knowledge. Such insights cannot be disregarded in discussions on the support needed for successful waste picker integration. This article will also show that most of the determinants mentioned by the WHO are relevant to waste pickers on the landfills covered in the underlying study.

To view the health risks of landfill waste pickers in a broader context, the article introduces and is guided by a socio-ecological framework.

### Theoretical Framework for the Study

**Figure 1:**
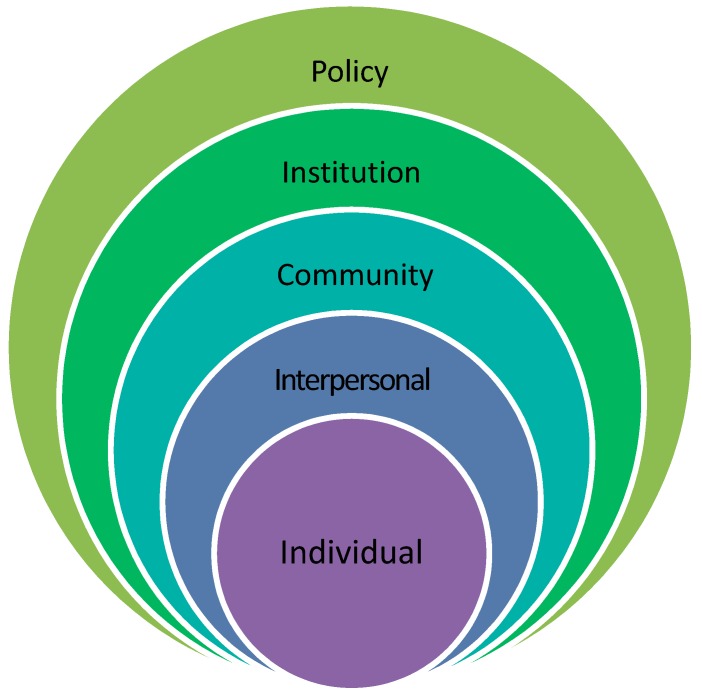
The socio-ecological framework. Source: Adapted from Bronfenbrenner (1999) [29].

The socio-ecological framework used in the article (illustrated in Figure 1) considers the complex interplay between individuals and their interpersonal relationships, the community, institutions and policies.

This framework is based on Bronfenbrenner’s ecological systems framework [29] which proposes that no person can be seen in isolation. Waste pickers and the health issues/risks they face should be viewed in the context of where they live and work [15]. To understand the depth and complexity of these health risks, one needs to understand the context in which waste pickers work and live as well as the role that institutions and policies play. Table 1, which has been adapted from The United Nations Children’s Fund (UNICEF) (2014), describes the levels in the framework [30]. 

In this article, the first three levels will be used to describe the results of the study and to offer insight into the health risks faced by waste pickers. Levels 4 and 5 will be used to frame the policy and practical recommendations and further research that need to be conducted. 

## 2. Methodology

As stated in the introduction, only two previous studies were conducted on the health status of the waste pickers in South Africa. These studies were micro-studies linked to a landfill in Durban and a landfill in Tshwane. To understand the health risks of the landfill waste pickers (LWPs) on the nine sampled landfill sites, a concurrent mixed-methods approach, with both qualitative and quantitative methodologies, was used for this exploratory research. A definition of mixed-methods research is provided by Creswell (2007:269) as: ‘… a procedure for collecting, analysing and mixing both quantitative and qualitative data at some stage of the research process within a single study to understand a research problem more completely’ [31].

In order to understand the health risks of the LWPs from a socio-ecological framework, qualitative interviews were firstly conducted with the landfill managers and officials on arrival in the town or at the landfill. The aim of the interviews was to understand the daily routine at the landfill and of the waste pickers, how the landfill is managed with the presence of the waste pickers, as well as the challenges and benefits of having the waste pickers on the landfill. Observations were made, relevant documents requested (such as rules for the LWPs) and permission was obtained to take photos of the landfill, its operations and facilities. 

Interviewing the waste pickers, the researchers collected both numerical and text-based information concurrently. The questionnaires, consisting of quantitative and qualitative questions were developed, piloted and used in different studies in South Africa, such as the study of Schenck and Blaauw (2011 a and b) [32,33] and Schenck et al. (2012) [34]. An adapted version was also used in the study by Viljoen (2014) on the street waste pickers in South Africa [8]. The findings were integrated into the analytical phase of the study [31]. 

Nine landfills in four of the nine provinces in South Africa were included in the sample.

- Three of the landfills were in cities/towns where extensive information was available from other studies on the health status of the population.
- Three landfills were selected on the basis that information from an earlier (2012) landfill waste pickers study [34] was available and comparisons could be made.
- Another two landfills were added following consultation with the Western Cape Government Department of Environmental Affairs (DEA)—Waste Management sector.
- Another rural landfill (BR), managed by the same municipality, was added to the research project after the completion of the fieldwork in the urban landfill (PR).

Before the fieldwork commenced, team members visited the sites to:

- assess the accessibility and security of the sites for researchers and fieldworkers;
- assess the suitability of the sites for satisfying the research aim;
- identify the necessary processes involved in obtaining permission from the municipalities/local authorities to conduct the study on the landfills;
- negotiate and obtain permission to enter the landfills;
- identify the language(s) spoken by the waste pickers on the landfills in order to recruit suitable fieldworkers who could speak the languages of the participants.

The reconnaissance phase also assisted in determining the sampling process to be followed with the waste pickers. The reconnaissance visits to the landfill sites revealed that a pre-planned sampling of the waste pickers was not viable due to the daily fluidity of the research population. The research team determined that the sample should be selected from those available and willing to participate, which represents an availability and convenience sampling technique and ensures that the sample is as representative as possible. Ethical clearance was obtained from the University of the Western Cape’s Senate Research Committee before the research commenced.

The researchers ensured that fieldworkers were recruited who were fluent in Afrikaans, English and each area’s own local language. IsiXhosa, Tswana, SeSotho and Ndebele were the dominant languages spoken on the visited landfills. Fieldworkers were trained in the data collection process before the visits to the landfills.

The fieldworkers were trained to administer the consent form and questionnaires in the language the participants understood best because some of the waste pickers had had limited schooling or no schooling at all. On all landfills, at least two researchers were present for the duration of the data collection process and could assist fieldworkers if problems arose. The data was collected during 2015 and 2016, with aspects such as seasonality being taken into account. For example, areas with very cold climates in winter were visited in spring or autumn to ensure the best possible coverage of the research population.

On arrival at a landfill, the researchers, in consultation with the landfill manager or operator, identified the best spot for the data to be collected. This spot was different for each landfill site. On some landfills the managers assembled all the waste pickers willing to be interviewed in front of the office at the entrance to the landfill, while on most of the landfills, the researchers proceeded to the designated area where the waste-picking activities took place. This was because the waste pickers did not want to leave their collected waste out of sight for fear of it being stolen and also so that they could be at the site when the trucks arrived to dump new waste. It was arranged with the waste pickers that, as soon as a truck entered the landfill, they could go and collect the recyclables and then return to complete their interview. 

Table 2 indicates the number of interviews conducted on each landfill. 

On all the landfills, the sample sizes were higher than 60%, except in PR where only 49% of the waste pickers present on the data collection day were interviewed. As can be seen in Table 2, landfill PR had the highest number of waste pickers present and was far larger than the other landfills visited. 

The quantitative data was captured in Excel and analysed using SPSS version 22 (IBM, Foster City, CA, USA). The qualitative data collected was analysed thematically. Observations and reflections from the fieldworkers provided contextual data, adding to the depth and richness of the study. 

## 3. Results

In this section, the results of the health risks experienced by the waste pickers are discussed in terms of the socio-ecological framework.

### 3.1. Level 1: Individual Level

Table 3. provides a breakdown of the biographical data of the waste pickers who were interviewed.

The racial composition was predominantly Blacks (80%) followed by Coloureds (20%) (According to the South African racial classification system, Coloureds refer to persons of mixed race). On most of the landfills, men were in the majority; on only two landfills were the majority of waste pickers women. One of the landfill sites, managed by a private waste management company, only allowed men on the site as it claimed that it was easier to manage if only one gender was present. The 2012 study conducted by Schenck et al. (2012) in the Free State also found slightly more men (52%) than women (48%) on the landfill sites [34]. The results of the latter study on landfills differed from those of the study conducted by Viljoen (2014) among street waste pickers in South Africa, where men were by far in the majority—mainly due to the risks involved for women [8]. 

Just over 42% of the respondents in the survey were younger than 35. The average age of the waste pickers was 39, with the youngest being 18 (No children under 18 were interviewed. Children were present on only one landfill and that was so that they could access glue, whose remnants could be found in a number of discarded bottles, for sniffing.) and the oldest 71. The median age was 38. In terms of South African legislation, the broad term ‘youth’ means people aged between 15 and 34. A significant percentage of LWPs, therefore, could be classified as youth. This is a reflection of the persistently high youth unemployment rate in South Africa which, according to StatsSA (2018), stands at 53% for the 15 to 35 age group [35]. 

Educational qualifications and literacy levels are regarded as important for the healthy functioning of individuals [7,36,37].

**Figure 2:**
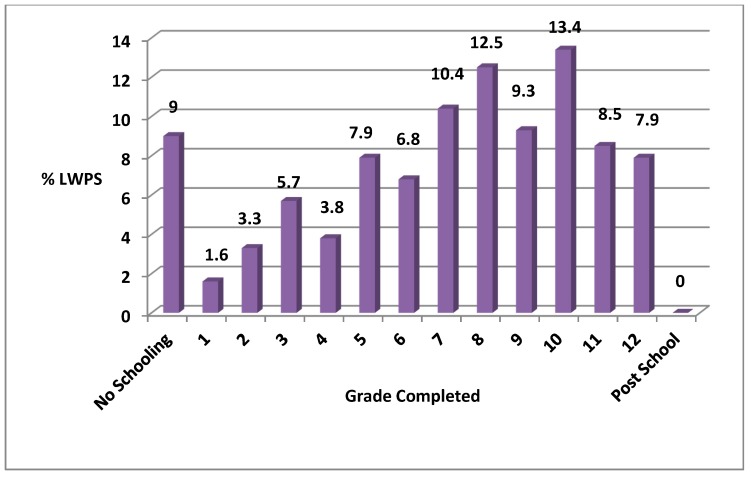
Highest qualification attained by landfill waste pickers (LWPs). Source: Survey data.

Figure 2 indicates the highest qualification attained by the LWPs participating in the study.

Of the total number (373) of respondents, 9% had no schooling, while 43.7% had obtained some secondary level education, ranging from Grade 8 to Grade 11, and less than 8% had completed matric (Matric or Gr 12 is the final year of the formal school system in South Africa). The results broadly corresponded with the findings of most other complementary studies in South Africa [8,32,33,38,39,40,41] which concluded that the educational attainment levels of waste pickers in the country were fairly low. On examination of the reasons for leaving school early, financial difficulties and family problems dominated. Family problems included one or both parents having died or fallen ill, the lack of community support and safety nets in the family, and the absence of an extended family had forced the respondents to search for work to secure the means to support them. Very few waste pickers left school early by choice. Instead, complex personal circumstances conspired to prematurely eject them from the formal education system. 

### 3.2. Level 2: Interpersonal and Social Risks Related to the Health and Safety of Waste Pickers 

The results showed that the biggest health and safety risks that the waste pickers faced were on a social and interpersonal level in their living and working spaces. Problems like stigmatisation, substance abuse, poverty, harassment and violence in their immediate environment were raised during the interviews [2,8,11,20,34]. Nevertheless, the waste pickers indicated that the landfill provided them with a sense of community. 

The majority (68%) of the waste pickers indicated that they worked together and helped each other. This was in contrast to the findings of a national study conducted among street waste pickers where only 27.7% of the sample indicated that they belonged to a group of recyclers who assisted each other [8]. When the landfill waste pickers were further prompted, they disclosed that they supported each other but did not work as a collective and did not share their income. They strongly emphasised their financial independence and the fact that they were self-employed, but helped each other in activities such as collecting and carrying recyclables. They also collected and cooked food together, lent money at times and cared for one another when sick. Previous studies [8,34,41] confirmed that waste pickers regarded themselves as self-employed but in some instances a strong sense of community prevailed on the landfills, despite the interpersonal conflicts and tensions that manifest as physical, psychological and social risks. These risks are summarised in Table 4.

The risks on an interpersonal level are closely linked to being part of a community. 

### 3.3. Level 3: Risks Associated with Being Part of A Community

Landfill waste pickers are part of a broader community than simply the waste-picking community. The LWPs are part of the waste picker community on the landfill with its officials, facilities or the lack thereof, the communities where they reside and they are in interaction with the broader municipality or landfill waste management company. This section contains information obtained from the LWPs and from the waste managers and officials, observations and photos taken.

According to Gutberlet and Uddin (2017), more than one-third of the world’s population lives in informal settlements with little or no basic services. Here open dumping, little or no waste collection, and contaminated water and soil are the norm [15]. Working on landfills and living in dire circumstances increase health risks. Many waste pickers lack access—either on the landfill or at home —to clean water, proper sanitation and a clean living environment [18]. Table 5 indicates how many of the waste pickers had access to basic amenities on the landfills.

A large group of the research population appeared to have some access to drinking water while they collected waste on the landfills. Most of the landfills had taps or tanks, but 80 (22%) of the waste pickers had no access to clean water. Most waste pickers (235 or 65%) indicated that they had access to food on the landfills. Half the waste pickers had no access to ablution facilities and needed to *‘go to the bushes’* because there were *‘no toilets here’* and *‘no showers here’*. The landfills with no facilities were also those where effective waste management of the landfill was absent. On one of the landfills, the landfill manager indicated that the municipality was put under administration, due to political infighting and mismanagement. The manager did not have a budget to implement good waste management, let alone having facilities available for the waste pickers. Not even the female official employed by the municipality to register incoming trucks had access to water and ablution facilities. There was no weighbridge or any infrastructure at the landfill except for a small dirty building in which the official could sit when it rains. 

Table 6 indicates the types of structures in which the waste pickers were sleeping. The results confirmed the finding of Cointreau (2006) and Jerie (2016) that waste pickers also lack access to basic facilities at home, which increased their health risks [16,18]. 

**Figure 3:**
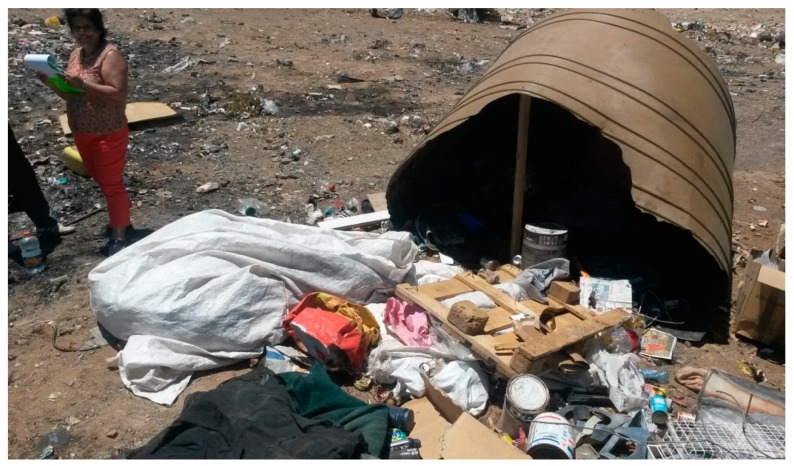
A ‘structure’ in which a waste picker sleeps on the landfill and which provides shade during the day.

If all formal structures were excluded, a significant proportion (59.9%) of the waste pickers slept in informal structures or in the veld or bushes or on the landfill (see Figure 3), which means that they lacked access to proper infrastructure, water or ablution facilities. Not having access to these facilities on both the landfill where they worked and at the place where they slept increased waste pickers’ vulnerability to health risks. 

Other risks to which the waste pickers were exposed while working on the landfills were mechanical, ergonometric, chemical, biological and environmental risks. These risks are discussed under level 3, as the health risks they experience relate to the management of the landfills.

#### 3.3.1. Mechanical Risks 

Mechanical risks refer to cuts, lacerations and bruises sustained in traffic/vehicle accidents, and fractures and other injuries due to landfill slides or uneven surfaces. The United Nations Environment Programme (UNEP) (2013) [1] reveals that a great deal of live ammunition can still be found on landfills in countries where there were or still are wars, such as Sudan, which constitute a major hazard for waste pickers because of the risk of explosion. According to Cointreau (2006) [16], waste pickers in developing countries are much more susceptible to the risk of getting cut because they have to scratch, often without protection, through mixed waste to get to the recyclables. Sorted waste, in contrast, limits the chance of injuries. 

**Figure 4:**
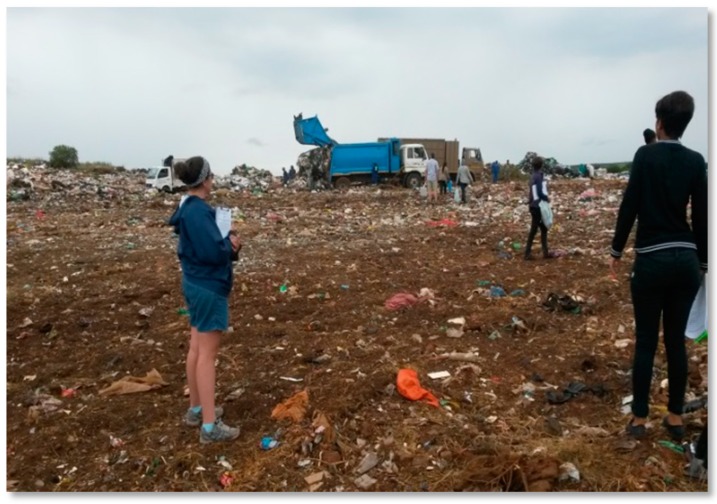
Separating man and machine on the landfill. Source: Authors.

On one of the landfills, the waste pickers did not make use of protective clothing such as gloves and masks, even when these were provided to them. On landfill ST, the officials will, every morning when the waste pickers sign in to enter the landfill, provide them with a reflective jacket for visibility and masks for protection. On the day of the research, the masks were hanging around the necks of the waste pickers, and it was not used for what it was meant for. It was also observed that on most of the landfills, the municipality was taking steps to try and separate the activities of trucks and people (see Figure 4), thereby lessening the risk of injury.

Despite efforts by some municipalities to ‘separate man and machine’, the waste pickers said that being run over by trucks was one of the biggest risks that they faced on the landfill sites. The researchers observed that three of the landfills (BN, PO and VR) employed ‘pointers’ to separate the trucks from the people. The pointers had the responsibility of ensuring that the trucks did not dump waste where the waste pickers were busy sorting an earlier load [42].

Even though various precautionary measures are taken by landfill operators, waste pickers are still routinely exposed to the risks cited by the respondents, which appear in Table 7. 

The type of work performed on the landfills poses risks that can only be avoided by keeping man and machine separate, wearing protective clothing and engaging in other forms of responsible waste management. The latter includes separation of material at source and introducing material recovery facilities to minimise risks [43]. What is also notable is the fierce competition that exists for valuable recyclables, which drives waste pickers to increase the risks that they are willing to take. This is a sign that LWPs view the opportunity cost of losing out on collecting recyclables as very high. On only one of the landfills did we find visible rules displayed, which were negotiated with the LWPs. The rules stipulated, among others, “*that no running after and climbing on vehicles will be allowed*”. According to the landfill official, these rules are strictly implemented. The LWPs are banned from the landfill for a few days, thereby forfeiting income, if they are found not adhering to these rules. 

#### 3.3.2. Ergonometric Risks

Ergonometric or musculoskeletal risks refer to damage from unnatural movements of the body [16,21]. In the context of waste picking, damage can be the result of hard physical work, such as pushing and pulling bags and trolleys and carrying large bags of recyclables.

**Figure 5:**
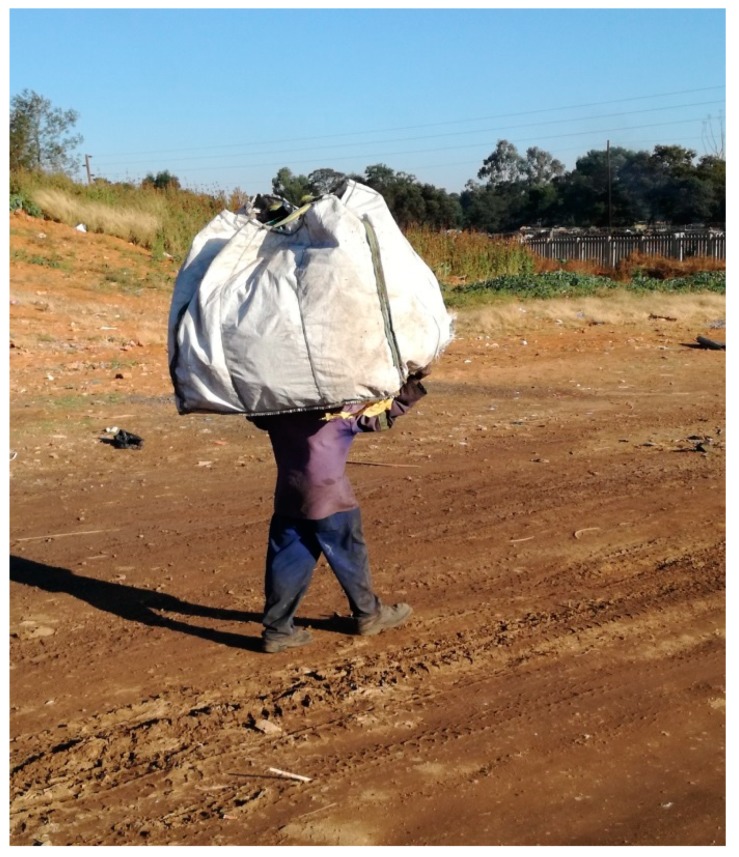
A waste picker carrying a heavy load of waste. Source: Authors.

As revealed in the literature [8,13,15,34], it is often said that waste picking has no barriers to entry, but this is not strictly true if one takes physical ability into account. Waste picking needs an able body but it can still lead to injury, particularly when it involves pushing heavy loads and pulling heavily laden trolleys over long distances [8] or carrying heavy bags (see Figure 5). 

It is interesting that very little mention was made during the interviews of back, leg or neck pains, despite the hard physical labour in which the LWPs were engaged. Rather, these conditions are more common among street waste pickers (SWPs) [8] as they have to walk longer distances and carry heavier loads. Only a few complaints were made by elderly waste pickers, in reference to the fact that they got tired of the hard, physical work: *‘The amount of work I have to put in; I am old and tired’* and the fact that they were getting *‘short of breath’*. They did, however, mention that they *‘help each other carry’*. It was earlier stated that 36% of the waste pickers assisted each other with the collection and carrying of waste and on all the landfills the buy back centres (BBCs) came to the landfill sites to buy the collected and sorted recyclable waste from the pickers. Different from the SWPs, they do not have to deliver it to the BBC. Adding to the ergonometric risks were the distances some of the LWPs had to walk to the landfills. At three of the landfills, the LWPs shared the distances they have to walk from where they live as a restraining factor. The VR landfill was previously next to the township and easily accessible. Then, because the landfill reached its capacity, a new landfill was built 5 km from the township. The LWP had to walk these distances daily to and from the landfill. No public transport was available for the waste pickers to use. Some indicated that on days when the BBCs came to fetch the recyclable waste, the BBC will offer them a lift. Similarly, the LWPs on the ST landfill previously lived on the landfill as their homes were far from the landfill. Moving them off the landfill, the municipality provided housing for some of the LWPs next to the landfill, but there were some who complained that they now have to walk more than an hour to the landfill as no transport is available at the time in the morning they leave home for the landfill or they do not have money to pay for transport. An additional risk that was created when the waste pickers moved off the landfill was linked to the *“first come first serve”* policy of the management. The municipality decided to limit the number of pickers on the landfill to enable better management of LWP. To be at the gate among the first 50 pickers, some said they had to leave home as early as 3 to 4am, increasing their safety risks. 

#### 3.3.3. Chemical Risks 

Jerie (2016) is of the opinion that diseases resulting from chemical and biological origins are often difficult to identify as occupational hazards as the diseases take time to manifest and there can often be multiple reasons for the diseases [18]. The origin can be the ill management of the landfill, the unhealthy environment where they reside, other people they are in contact with, and a lack of knowledge on how to deal with certain chemicals and waste. 

Chemical risks are regarded as gases, pressurised gases, hazardous chemicals and air pollution, which can result in respiratory problems, skin problems and neurological and kidney and liver diseases [18]. Mothiba et al. (2017) point out that gases and smoke can emanate from the burning of waste, either as a form of waste management or to retrieve the metal parts embedded in electronic waste, for example [24]. They can also be in contact with hazardous waste when opening tins and buckets that contained hazardous substances. 

The waste pickers mentioned a number of other health risks related to respiratory problems and substance abuse (see Table 8).

On one of the landfills, a shoe factory had discarded glue in Polyethylene Terephthalate (PET) bottles which was being sniffed by young children.

The substances obtained on the landfills were less seen as a chemical hazard as that it created problems among the LWPs. In the office of the PO landfill, the following rule was negotiated with the LWP: *No drugs are allowed on site neither is anyone intoxicated by such allowed on site.* The rule was strictly implemented. 

#### 3.3.4. Biological Risks

Biological risks faced by waste pickers on the landfills refer to infections resulting from consuming bad food and drinking polluted water, and coming into contact with faecal matter, blood, bodily fluids, animal flesh, and dead and live infected animals such as rodents [2,16,18,26]. 

Aspects not mentioned by the waste pickers were the particular illnesses they were susceptible to. Tuberculosis was mentioned by several waste pickers, but Hepatitis B was not, despite being regarded by many researchers as one of the easiest illnesses to contract [16,21]. One possible reason for this is that waste pickers are probably not aware of, nor tested or treated for, these illnesses. The need for medical tests and care is of importance to the waste pickers. However, if not desperately ill, they might choose not to visit a medical facility so that they do not forfeit the opportunity of earning a daily income.

**Figure 6:**
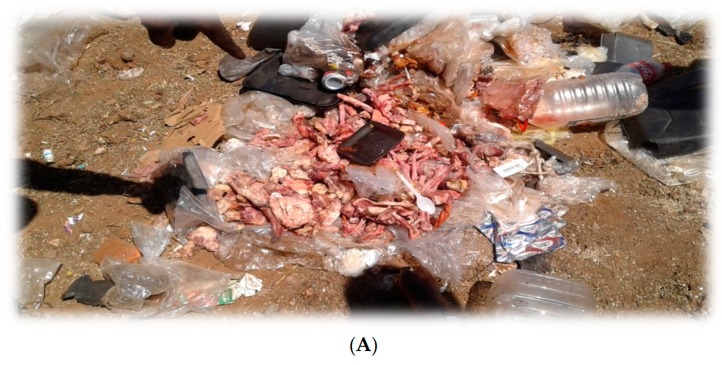

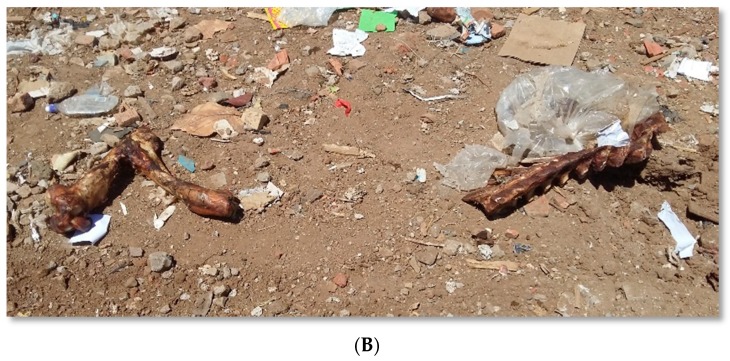
Chicken meat (**A**) and beef carcasses (**B**) discarded on landfills. Source: Authors.

In this and other studies, waste pickers acknowledged that they ate from the dump [1,7,20]. The biological risks mentioned in the current study are listed in Table 9, with illustrations in Figure 6A,B.

Drinking polluted water from the landfills is a potent reminder that clean water is not always available (see Table 5). Furthermore, eating food from the landfills highlights the desperate poverty levels of some waste pickers. As indicated in Table 10, in total, 184 (or 50.8%) of the responding 362 LWPs reported that they collected food from the landfills. The landfill with the highest percentage of waste pickers who obtained food from the landfill either for themselves or their families was OU (90.9%), followed by ST (78.6%) and PO (64.7%). The day when data was collected on landfill OU, it was observed that the landfill is much more than just a landfill where recyclables are collected. The landfill is opposite the township and during the day, many residents from the township came over to the landfill to collect wood, household goods and food. During the afternoon after school, many mothers and their children also walked into the landfill looking for food. During the discussion with the landfill manager, he was aware of this situation and sympathetic towards the poverty stricken community.

To avoid the illnesses associated with eating contaminated food, the waste pickers only ate what looked right to them. *‘I collect the things I see and look right.’*

In total, 125 waste pickers shared with the researchers that there were days when they did not have food to eat, while 11 indicated that at times they went without food for up to 10 or even more days per month. It seems that these days might have been mostly over weekends when they could not collect and sell recyclables to buy food or access food from the landfill. On further probing, some indicated that they had a safety net in the form of social grants, family/neighbours, the ‘army’, and bakeries, shops and churches that supported them with food. 

It also seemed that the management of soiled nappies was becoming increasingly important. The GBCSA (2018) [44] estimates that the number of soiled disposable nappies ending up on landfills exceeds 3.5 billion per year, or close to 1.1 million tonnes per year worldwide [44]. 

Particularly alarming in the South African context, which was not mentioned in the other literature, was the discovery by the waste pickers of aborted babies. Not only do decomposed bodies present significant health risks, it is mainly an emotionally upsetting experience ‘… *when babies are found between rubbish’* (cried one respondent) and ‘*… also the trauma associated with it’* (said another respondent). The landfill managers and officials indicated that if they are made aware of such a discovery, the police will be notified. This tragedy is an aspect that needs further attention from researchers. 

#### 3.3.5. Environmental Risks

**Figure 7:**
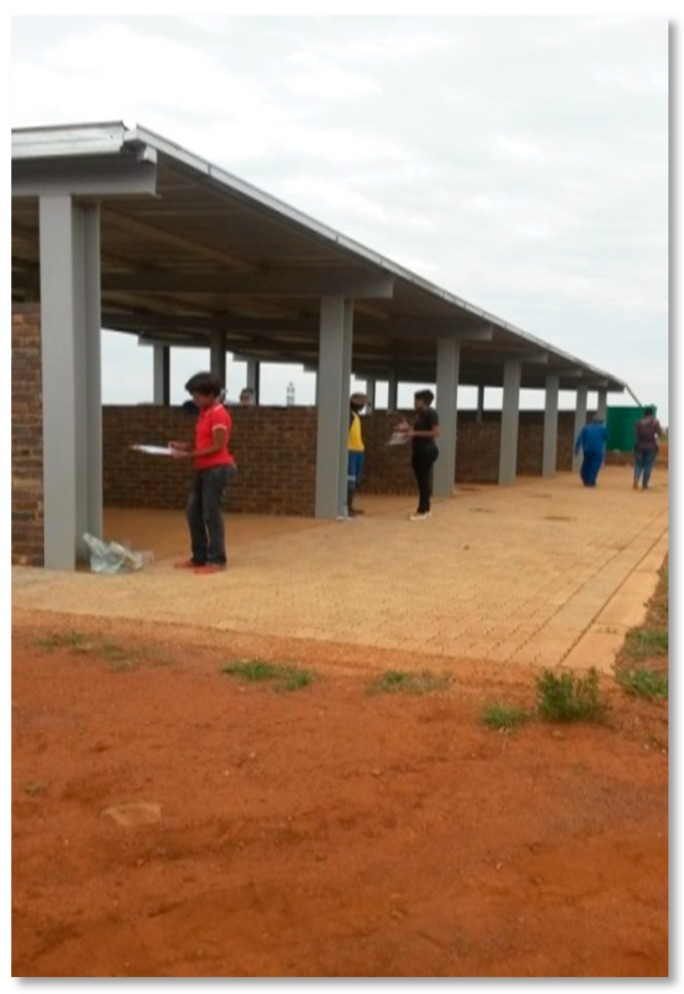
Shade provided on a landfill. Source: Authors.

The researchers observed that waste pickers on only one landfill (VR) had access to shade and shelter against the sun, cold weather and rain as shown in Figure 7.

**Figure 8:**
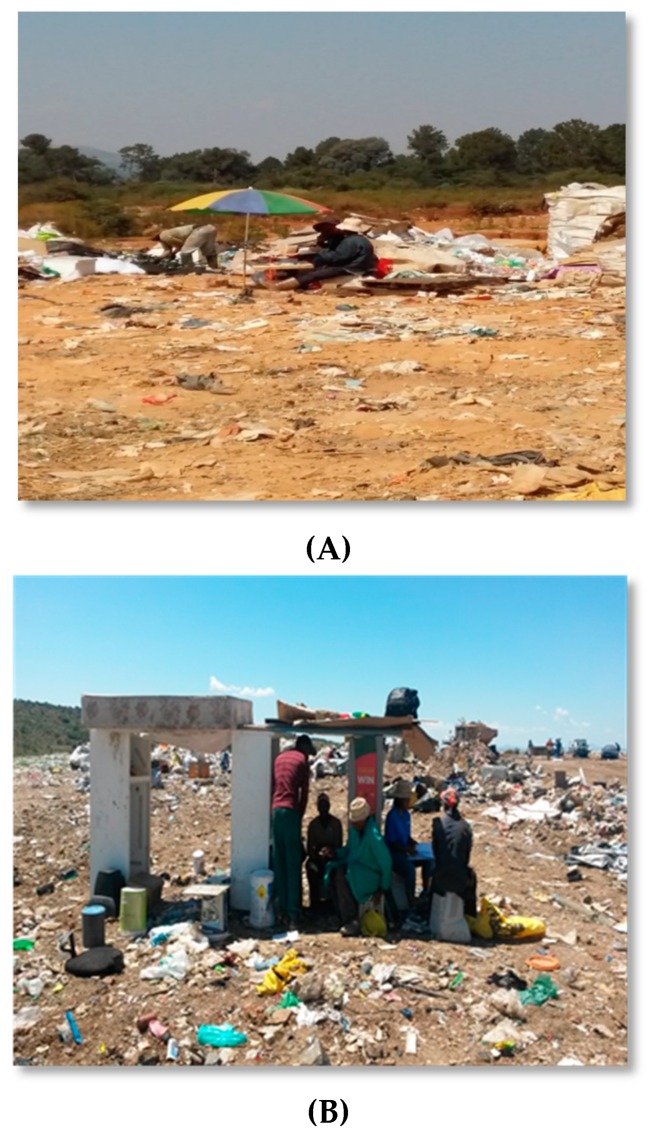
(**A,B**) Different types of shelters on landfills. Source: Authors.

On some landfills, waste pickers had built their own structures to provide some shelter against the elements (see Figure 8A,B).

Exposure to the elements and extreme weather, with little or no protection, was a major problem cited by the waste pickers as it not only negatively affected their health but also their income. Comments included: *‘Extreme cold and heat without having access to shade or protection’, ‘The smell and the sun’ and ‘It gets hot and I become disorientated’.*

## 4. Conclusions

Taylor and Triegaardt (2018) are of the opinion that a core feature of transformative social welfare policy is to move away from the perception that people are exploited and blamed; rather, focus on their circumstances (looking through both a macro and micro lens) and address the factors that cause the deprivation by implementing programmes and other interventions that can improve their quality of life [45]. These policies and programmes should be embedded in human rights and social justice. Reflecting on the health risks of the waste pickers, urgent measures should be taken to improve their quality of life and address the prevalent social injustices.

The health risks and general well-being of waste pickers on the landfills directly relate to Levels 4 and 5 of the socio-ecological framework and encompass the institutions and multilevel government (national, provincial and local) policies that have the power to favourably impact and improve the health and socio-economic circumstances of the waste-picking community.

Level 4 deals with the institutions that influence the day-to-day functioning of the LWPs. This study has shown that, with a few exceptions, very little effort has been made by any level of government—and particularly local government—to take an interest in and provide for the health and safety of the waste pickers in the form of protective clothing, shade, medical testing and other basic facilities that would ensure a reasonable level of comfort and dignity. 

At the international level, with the adoption of the 2015 Sustainable Development Goals (SDGs) [46], world leaders have pledged to transform the working environment and leave no-one behind. At the national level, South Africa’s Constitution promises clean and safe environments for all people. In South Africa, the National Environmental Management: Waste (NEM:WA) Act 59 of 2008 [47] specifies the environmental and health standards that each landfill operator or permit holder must adhere to for the proper management of waste and, specifically for the purpose of this article, the protection of those working and residing on landfills. The environmental right, as stated in section 24 of the Constitution, is: ‘… everyone has a right to an environment that is not harmful to their health or well-being…’ The South African Human Rights Commission embellishes this as follows: ‘The Constitution further places an obligation in terms of section 152 (1) (b) and (d) on the part of local government as stipulated in sections 4(2) (d) and 4(2) (i), 73(1) and (2) of the Municipal Systems Act 32 of 2000 to ensure that the right to a clean and healthy environment is fulfilled.’ If policies are to be transformational, reforms should be implemented that fundamentally change social institutions and relations to bring about greater inclusivity and a more equitable distribution of economic power and resources.

Given the valuable role played by informal waste pickers in the recycling industry and the broader waste economy [7], the time has come to take steps to at least reduce the health risks that they face and to enhance their dignity and well-being, starting with providing them with access to basic amenities. Gutberlet and Uddin (2017), Cointreau (2006) and UNEP (2013) recommend that a comprehensive health plan be developed for informal waste pickers, which would include mapping and addressing the possible risks that they face on the streets and landfills [1,15,16]. Such a plan should incorporate a proactive and inclusive approach to waste pickers’ health and safety, including the provision of vaccinations, protective clothing and access to medical care, and basic facilities such as water and sanitation and pest control on the landfills.

UNEP (2013) makes further recommendations, such as controlled access onto landfills to prevent fights and gangsterism, and a code of conduct drawn up together with the waste pickers [1]. To ensure easier and safer access to waste, UNEP (2013) recommends the introduction of a waste-sorting facility and toolkit [1]. Staff and site officers from the company or government entity that manages the landfill should have a presence on the landfill at all times. Gutberlet and Uddin (2017) add to these suggestions, saying that efforts should be made to induce behavioural change in the community towards safe recycling, sorting and separation and, above all, sound waste management [15].

Integrating the self-employed waste pickers into the formal waste management system should be comprehensive in order to limit health risks. Waste pickers will never have a risk-free environment, but the right policies and supportive institutions can help to mitigate these risks and create a more sustainable and dignified working environment.

A final recommendation for the next step of the research process is to use the results of this research to determine quantitatively the depth and extent of the health risks of the waste pickers to be able to map and plan future health plans and practices as part of the integration process. 

## Figures and Tables

**Table 1:** Description of levels in the socio-ecological framework.

Level	Description
**Individual**	Characteristics of an individual, such as gender, age, racial/ethnic identity, sexual orientation, literacy, and others.
**Interpersonal**	Formal (and informal) social networks and social support systems in which individuals function (such as family, friends, peers and co-workers) and the effects thereof.
**Community**	Relationships among organisations, institutions and informational networks within defined boundaries, including the built environment, community leaders, businesses and transportation.
**Institutional/Organisational**	Organisations or social institutions, with operational rules and regulations affecting how, or how well, work is performed.
**Policy/Enabling Environment**	Local, state, national, and global laws and policies, and access to health and social services, and/or shortcomings in these respects.

Source: Adapted from UNICEF (2014) [30].

**Table 2:** Number of interviews conducted on each landfill *.

Landfill	Number of Waste Pickers Interviewed	Estimated Number of Waste Pickers on Site on the Day of the Interviews	Percentage of Waste Pickers Interviewed on Each Landfill
ST	46	50	92%
OU	32	50	64%
BN	38	60	63%
BS	49	60	81%
BO	39	40	97%
PR	98	200	49%
BR	31	40	77%
VR	23	30	76%
PO	17	20	85%
**Total**	**373**	**570**	

* The names of the landfills are listed as acronyms in the interests of anonymity. Source: Survey data.

**Table 3:** Profile of the waste pickers interviewed.

Category	Profile of the Waste Pickers
Race	Black: 80%
	Coloured: 20%
Age	Average age: 39
	Median age: 38
	Youngest: 18
	Oldest: 71
	Under 35 years (classified as youth in South Africa): 42%
Gender	Men: 60%
	Women: 40%

Source: Survey data.

**Table 4:** Interpersonal and social risks on the landfills as experienced by waste pickers.

Racial Tension	*‘There is a racial tension between Coloureds and Blacks.’*
	*‘The Makwerekweres* (referring to Zimbabweans) *steal our waste.’*
Fighting	*‘Arguments about waste brought to the site and the dominance of the males over us females.’*
	*‘Swearing and arguments and sometimes the way people treat each other (pushing when they see goods).’*
	*‘Fighting. Friend has been stabbed to death.’*
	*‘People stab each other and machines run people over.’*
Insults	*‘We get insulted.’*
	*‘People insult each other and lack respect for older people.’*
Theft	*‘Outside people steal our boxes.’*
	*‘Crime—young boys steal our cell phones and wallets.’*
	*‘Crime is too much—they steal from us the tsotsis* (gangsters).*’*
Physical danger	*‘It is nice but dangerous.’*
	*‘We* (women) *get robbed and raped.’*
	*‘Skollies* (gangsters) *are dangerous.’*
	*‘Arguments, fights and knife attacks.’*
	*‘Safety is a problem when we have to walk home.’*
Substance abuse	*‘When we are drunk we get injured easily,’*
	*‘Some are intoxicated on site and run the risk of being run over by the machines.’*
	*‘“Nayope”* (a cheap drug) *boys here causing trouble.’*
	*‘Cheating and stealing and having no respect for each other.’*

Source: Survey data.

**Table 5:** Access to basic amenities on the landfills.

Basic Needs	No	Yes	Total
Drinking water	80	284	364
Food	126	235	361
Toilet	180	182	362
Place to wash yourself	198	98	296

Source: Survey data.

**Table 6:** Types of structure in which landfill waste pickers sleep (*n* = 364).

Sleeping Structure	*n*	%
Construction site	4	1.1
Backyard room/backyard shack/wendyhouse	29	7.9
Veld/bushes	1	0.3
Shack	177	48.5
Hostel/shelter	6	1.6
House (bricks/reeds, etc.)	139	38.1
Buy-back centre/depot	1	0.3
Bungalow on municipal grounds	2	0.5
On landfill site	4	1.1
Mud house	1	0.3
Boxes and bricks	1	0.3
**Total**	**365**	**100**

Source: Survey data.

**Table 7:** Risks to which waste pickers are exposed.

Glass	*‘Yes, get cut by glass if I do not put gloves on.’*
	*‘Injured sometimes with bottles.’*
	*‘Walk on glass and get injured.’*
Vehicles	*‘Injuries to feet or getting knocked down by a truck.’*
	*‘There is usually a stampede when the trucks arrive.’*
	*‘Risk of tractors moving dirt and being run over.’*
	*‘Trucks driving fast on site.’*
	*‘Pickers rush toward in-coming trucks and so injure themselves.’*
	*‘Maybe hurt by trucks, friend was run over by truck. Sustained head injuries.’*
	*‘Having to chase the bakkie* (pick-up) *to get something. They jump on the bakkie to get to the recyclables first).’*
Sharp objects like nails, needles and wires	*‘Cutting your fingers or stepping on a nail.’*
	*‘You can cut your fingers when searching for waste.’*
	*‘Can cut or lose a finger.’*
	*‘Wire cuts.’*
	*‘Needles from the hospital.’*
Landfill surface	*‘Danger of falling in holes where we are working.’*
	*‘I broke a bone (leg).’*

Source: Survey data.

**Table 8:** Chemical-related health risks mentioned by waste pickers.

Respiratory problems	*The weather and pollution.’*
	*The dust, polluted air, inhalation of chemicals, rotten objects, the cold in winter.’*
	*‘We cough a lot because of dust and smells.’*
Diminished capacity substance abuse	*‘Glue sniffing.’*

Source: Survey data.

**Table 9:** Biological risks mentioned by waste pickers.

Tuberculosis	*‘Illness caused by contracting TB,*
	*‘Air pollution and dust that can lead to sicknesses such as TB.’*
	*‘Chest problems and TB*
Spoilt food	*‘Coming into contact with or eating contaminated/rotten food, it makes us ill as we do eat bad food from site.’*
	*‘When you pick up rotten food you can get sick.’*
	*‘Eating anything that can be bad for you.’*
Polluted water	*‘When you eat or drink poisons and the water pollution.’*
	*‘Drinking polluted water.’*
Soiled nappies	*‘Soiled nappies.’*
	*‘I hate working through smelly nappies.’*
Dead babies	*‘Aborted babies.’*
	*‘Get dead babies and a lot of them.’*
	*‘Opening plastics and finding stillborn babies in them.’*
	*‘Picking up babies.’*
	*‘Sad things that they throw here* (referring to babies)*.’*
	*‘I do not like picking up foetuses.’*
Animals/insects	*‘Snakes, worms.’*
	*‘And snake bites.’*
	*‘The place is full of mice and I am afraid of them.’*
	*‘Fleas.’*
Rotten meat/waste	*‘The smell of dead animals.’*
	*‘Everything is thrown here, so we get sick and the smell is not good.’*

Source: Survey data.

**Table 10:** Number of waste pickers collecting food from the landfill (*n* = 362).

Landfill Site	Yes		No		Total	
	*n*	%	*n*	%	*n*	%
**BN**	19	48.7	20	51.3	39	100
**BS**	28	57.1	21	42.9	49	100
**BO**	12	40	18	60	30	100
**BR**	16	50	16	50	32	100
**PR**	33	34.7	62	65.3	95	100
**OU**	30	90.9	3	9.1	33	100
**PO**	11	64.7	6	35.3	17	100
**ST**	33	78.6	9	21.4	42	100
**VR**	2	8.3	22	91.7	24	100
**Total**	184	50.8	177	48.9	361	100

Source: Survey data.
